# Sepsis in der prähospitalen Notfallmedizin: Was können prähospital Tätige aus der Surviving Sepsis Campaign und S3-Leitlinie ziehen?

**DOI:** 10.1007/s10049-021-00949-y

**Published:** 2021-11-17

**Authors:** Manuel Obermaier, Markus A. Weigand, Erik Popp, Florian Uhle

**Affiliations:** Klinik für Anästhesiologie, Universitätsklinikum Heidelberg, Im Neuenheimer Feld 420, 69120 Heidelberg, Deutschland

**Keywords:** Fokus, Septischer Schock, Infektion, Rettungsdienst, Antiinfektiva, Focus, Septic shock, Infections, Emergency medical services, Antiinfectives

## Abstract

**Hintergrund:**

Die Sepsis ist eine notfallmedizinische Herausforderung – denn diese lebensbedrohliche Organdysfunktion, verursacht durch eine dysregulierte Wirtsantwort auf eine Infektion, präsentiert sich in vielfältiger Ausprägung und wird deshalb häufig erst zu spät erkannt.

**Fragestellung:**

Die kürzlich publizierten „surviving sepsis campaign“-Guidelines und die deutsche S3-Leitlinie geben Empfehlungen zur Diagnostik und Therapie der Sepsis im intrahospitalen bzw. intensivmedizinischen Setting, gehen jedoch nicht explizit auf den Bereich der prähospitalen Notfallmedizin ein. Ziel der Arbeit ist es, die Evidenzlage im Hinblick auf die prähospitale Versorgung von Patienten mit Verdacht auf Sepsis herauszuarbeiten und daraus Handlungsoptionen für den Notarzt- und Rettungsdienst abzuleiten.

**Diskussion:**

Die Therapie der Sepsis und des septischen Schocks wird in Bündeln zusammengefasst, wobei das erste idealerweise innerhalb der ersten Stunde abgeschlossen sein soll – analog zum Konzept der „golden hour“ bei anderen notfallmedizinischen Entitäten wie der Traumaversorgung. Die prähospitale Therapie fokussiert sich auf die Sicherung der Vitalparameter gemäß ABCDE-Schema, wobei insbesondere der Volumentherapie ein hoher Stellenwert zukommt. Die weiteren Maßnahmen des „1 h bundle“, wie Laktatmessung, Gewinnung mikrobiologischer Proben und Beginn einer antiinfektiven Therapie, sind regelhaft erst in der Klinik möglich. Ziel ist eine schnellstmögliche Fokussanierung, wofür die Auswahl und Vorabinformation einer geeigneten Zielklinik zur Initiierung und Bahnung der weiteren klinischen Diagnostik- und Behandlungspfade, eine strukturierte und gezielte Übergabe sowie regelmäßige Fortbildung erforderlich sind.

## Abstract (en)

**Background:**

Sepsis is a challenge in emergency medicine, as this life-threatening organ dysfunction, caused by a dysregulated host response to an infection, presents manifold and therefore is often recognized too late.

**Objectives:**

Recently published surviving sepsis campaign and German S3 guidelines provide recommendations for diagnosis and therapy of sepsis in an in-hospital or intensive care setting, but do not particularly address out-of-hospital emergency medical care. We aim to work out the evidence base with regard to the out-of-hospital care of patients with suspected sepsis and to derive treatment recommendations for emergency medical services.

**Conclusions:**

Therapy of sepsis and septic shock is summarized in bundles, whereby the first bundle should ideally be completed within the first hour—in analogy to “golden hour” concepts in other emergency medical entities, such as trauma care. In the out-of-hospital setting, therapy focuses on securing vital parameters, according to the ABCDE scheme, with a particular focus on volume therapy. Further procedures within the 1 h bundle, such as lactate measurement, obtaining microbiological samples, and starting an anti-infective therapy, are broadly available in hospital only. The aim is to control the site of infection as soon as possible. Therefore, an appropriate designated hospital should be chosen carefully and informed in advance, in order to initiate and pave the way for further clinical diagnostic and treatment paths. Moreover, structured and target-oriented handovers, as well as regular training, are required.

## Hinführung zum Thema

Die Sepsis ist mit einer Inzidenz von jährlich rund 50 Mio. Neuerkrankungen weltweit eine der Haupttodesursachen. Damit wird gemäß der „Global-burden-of-diseases“-Studie jeder fünfte Todesfall durch eine Sepsis verursacht [35]. Der Verlauf gestaltet sich mitunter fulminant und führt zu einer hohen Sterblichkeit [39]. Sowohl die Inzidenz als auch die Sterblichkeit der Sepsis ist deutlich höher als die anderer Notfälle wie z. B. Myokardinfarkt oder Schlaganfall [41].

### Definition.

Sepsis ist definiert als akut lebensbedrohliche Organdysfunktion, die verursacht wird durch eine dysregulierte, inadäquate Wirtsantwort auf eine Infektion [9, 43].

## Sepsis in der prähospitalen Notfallmedizin

In der Notfallmedizin stellt die Sepsis eine besondere Herausforderung dar, da sich das Syndrom Sepsis in vielfältiger Ausprägung und zunächst nur wenig spezifisch präsentieren kann und aus diesem Grund häufig nicht oder zu spät erkannt wird [23]. Obgleich die Sepsis bereits seit 2016 zu den Tracer-Diagnosen gehört [16], bleibt anzunehmen, dass viele Patienten, die mit vermeintlich „schlechtem Allgemeinzustand (AZ)“ unter dem Stichwort „AZ-Verschlechterung“ über den Rettungsdienst in ein Krankenhaus eingeliefert werden, in Wirklichkeit eine beginnende oder manifeste Sepsis aufweisen.

Die vergangenes Jahr publizierte S3-Leitlinie zur Prävention, Diagnose, Therapie und Nachsorge der Sepsis legt den Fokus auf das intensivmedizinische Setting und geht nicht explizit auf die Besonderheiten der prähospitalen Versorgung von Patienten mit Verdacht auf eine Sepsis ein [9]. Deshalb möchten die Autoren die Gelegenheit nutzen, um die aktuelle klinische Evidenzlage explizit im Hinblick auf die prähospitale Notfallversorgung herauszuarbeiten und daraus Handlungsoptionen für den Notarzt- und Rettungsdienst abzuleiten.

## Einsatztaktik und Zeitmanagement

Das klinische „1 h bundle“ sollte idealerweise innerhalb der ersten Stunde abgeschlossen sein. Trotz fehlender harter Evidenz für die Festlegung eines Zeitpunkts von 60 min nach dem medizinischen Erstkontakt [17] folgt diese Logik dem Prinzip einer „golden hour“, wie es bei vielen anderen notfallmedizinischen Entitäten, wie beispielsweise dem Polytrauma, Schlaganfall oder okklusiven Myokardinfarkt, etabliert und akzeptiert ist [9, 12, 15, 16].

**Figure 1:**
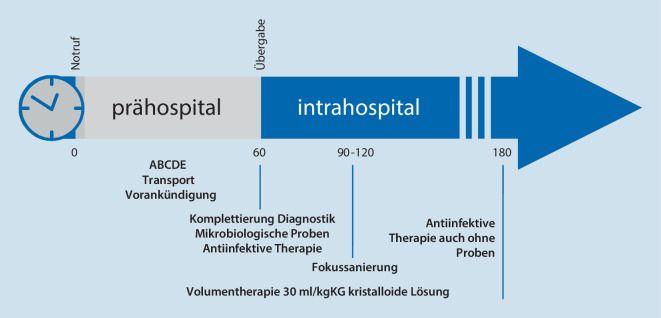

Für die prähospitale Versorgung folgt daraus, dass Patienten mit (Verdacht auf) Sepsis nach höchstens 60 min in einem geeigneten Krankenhaus aufgenommen werden sollen. Dazu gehören nach Überzeugung der Autoren eine über 24 h besetzte Notaufnahme mit Verfügbarkeit der entsprechenden Fachgebiete (prinzipiell innere Medizin und Chirurgie sowie ggf. Neurologie, Pädiatrie etc.), eine 24-h-Verfügbarkeit von Labor (Point-of-Care-Testung, zeitnahe Notfallwerte, Erregerdiagnostik) und Bildgebung (insbesondere Computertomographie) sowie eine freie Intensiv- und ggf. Operationskapazität. Die Diagnostik einschließlich der mikrobiologischen Probenentnahme sollte innerhalb von 90 min nach Notrufeingang erfolgen (Abb. 1, [16]).

## Diagnosekriterien

Sollte sich aus den Angaben des Patienten bzw. der Angehörigen der Verdacht auf das Vorliegen einer Infektion ergeben, sollte dieser durch eine sorgfältige körperliche Untersuchung erhärtet werden. Allerdings ist eine umfassende klinische Untersuchung an der Einsatzstelle häufig bereits aufgrund äußerer Umstände limitiert. Somit kann eine präzise Untersuchung meist erst in der Notaufnahme durchgeführt werden; diese muss dann fundiert erfolgen. Da der Rettungs- und Notarztdienst über die Untersuchung hinaus regelhaft Eindrücke von den (häuslichen) Umgebungsbedingungen erhält, sind Hinweise zum vermuteten Fokus für die Notaufnahme durchaus hilfreich und willkommen – diese dürfen aber nicht zu einem Fixierungsfehler führen.

Daneben treten bei der Sepsis häufig unspezifische Symptome des Gesamtorganismus, wie Fieber, Schüttelfrost, Hyperventilation, Vigilanzminderung, und unspezifische Schockzeichen, wie Tachykardie und Hypotension, oder allgemeines Krankheitsgefühl bis hin zu einem schwerstkranken Gesamteindruck („Facies hippocratica“) auf.

Abhängig vom jeweiligen Fokus sind weitere charakteristische Symptome möglich, als Merkhilfe eignet sich das Akronym „LUCCAASS“ (Tab. 1, [24]).

**Table 1:** 

Fokus	Symptome	Häufigkeit^a^ in %
**„L**ung“ (Lunge)	Dyspnoe, trockene Rasselgeräusche, Husten, Sputum, Zyanose, atemabhängiger Brustschmerz	51–63
**„U**rine“ (Urogenitalsystem, Niere)	An‑/Oligurie, Schmerzen in Blase oder Flanke, Klopfschmerz Nierenlager, trüber oder übelriechender Urin	2–18
**„C**entral nervous system“ (zentrales Nervensystem)	Kopfschmerz, Vigilanzstörung, Fieber, Meningismus, Lichtempfindlichkeit, Übelkeit, Erbrechen, Krampfanfall	1–3
**„C**ardiac“ (Herz)	Neues Herzgeräusch, Fieber, Rhythmusstörungen, Hinweise aus Anamnese und Untersuchung auf kardiale Risikofaktoren, degenerative Herzklappen, Operation, i. v.-Drogenabusus, einliegende Katheter, Dialyse, rheumatisches Fieber	1–2
**„A**bdomen“ (Gastrointestinaltrakt, Abdomen)	Bauchschmerz, Abwehrspannung, Blumberg-Zeichen, hochgestellte oder fehlende Darmgeräusche, Obstipation, Übelkeit, Erbrechen, Diarrhö, Hinweise aus Anamnese bzw. Untersuchung auf Operation, Immunsuppression	10–41
**„A**rthritis“ (Gelenke, Implantate)	Schmerz, Funktionseinschränkung, Hinweise aus Anamnese bzw. Untersuchung auf Operation (z. B. Narben, Prothesenausweise), Immunsuppression, Rheumatoide Arthritis	1–2
**„S**kin“ (Binde- und Stützgewebe, Haut)	Sichtbare Wunde, Rötung, Schwellung, Schmerz, Überwärmung, Funktionseinschränkung	7–10
**„S**pine“ (Wirbelsäule)	Myalgien, Rückenschmerzen, periphere neurologische Defizite (Parästhesien, Paresen)	k. A.
(Primäre Bakteriämie)	Unspezifisch	2–7

*k. A.* keine Angabe

^a^Die Häufigkeitsangaben nach [13, 14, 20, 39] beziehen sich auf Intensivstationen in Deutschland. Da nicht jeder Patient mit Sepsis aus der Notaufnahme auf eine Intensivstation verlegt wird, lassen sich die Häufigkeiten nicht uneingeschränkt auf das prähospitale Setting [5] übertragen

Früher wurde zur Diagnosestellung einer Sepsis neben den Hinweisen auf eine Infektion das Konzept des „systemischen inflammatorischen Responsesyndroms (SIRS)“ [8, 22] mit den Kriterien

- Hypo‑/Hyperthermie,
- Tachykardie,
- Hyperventilation oder Horovitz-Quotient < 200,
- Leukozytose/-penie oder > 10 % unreife Leukozyten

herangezogen. Auch wenn anhand der SIRS-Kriterien 12,1 % aller Patienten mit Sepsis nicht als solche erfasst werden [19], sollte die Symptomkonstellation im Hinterkopf behalten werden.

In der aktuellen Sepsis-3-Definition [43] wurden die SIRS-Kriterien durch den „Sequential-organ-failure-assessment(SOFA)“-Score (Tab. 2, [48]) abgelöst. Sollte dieser Score um ≥ 2 Punkte ansteigen, kann die Diagnose „Sepsis“ gestellt werden. Andernfalls sollten zunächst Differenzialdiagnosen abgeklärt und bei fortbestehendem Verdacht eine mögliche Sepsis reevaluiert werden [43].

**Table 2:** 

Organsystem	0 Punkte	1 Punkt	2 Punkte	3 Punkte	4 Punkte
*Atmungssystem*					
Horovitz-Quotient	≥ 400 mm Hg (≥ 53,3 kPa)	< 400 mm Hg (< 53,3 kPa)	< 300 mm Hg (< 40 kPa)	< 200 mm Hg (< 26,7 kPa)	< 100 mm Hg (< 13,3 kPa)
*Gerinnung*					
Thrombozyten	≥ 150/nl	< 150/nl	< 100/nl	< 50/nl	< 20/nl
*Leber*					
Bilirubin	< 1,2 mg/dl (< 20 µmol/l)	1,2–1,9 mg/dl (20–32 µmol/l)	2,0–5,9 mg/dl (33–101 µmol/l)	6,0–11,9 mg/dl (102–204 µmol/l)	≥ 12,0 mg/dl (≥ 204 µmol/l)
*Kardiovaskuläres System*					
Katecholamine (Laufrate über ≥ 1 h)	MAP ≥ 70 mm Hg	MAP < 70 mm Hg	Dopamin ≤ 5 µg/kgKG und Minute *oder* Dobutamin (dosisunabhängig)	Dopamin 5,1–15 µg/kgKG und Minute *oder* Adrenalin ≤ 0,1 µg/kgKG und Minute *oder* Noradrenalin ≤ 0,1 µg/kgKG und Minute	Dopamin > 15 µg/kgKG und Minute *oder* Adrenalin > 0,1 µg/kgKG und Minute *oder* Noradrenalin > 0,1 µg/kgKG und Minute
*Zentrales Nervensystem*					
GCS	15	13–14	10–12	6–9	< 6
*Niere*					
Kreatinin	< 1,2 mg/dl (< 110 µmol/l)	1,2–1,9 mg/dl (110–170 µmol/l)	2,0–3,4 mg/dl (171–299 µmol/l)	3,5–4,9 mg/dl (300–440 µmol/l)	≥ 5,0 mg/dl (≥ 440 µmol/l)
Diurese	–	–	–	< 500 ml/d	< 200 ml/d

*GCS* Glasgow Coma Scale, *MAP* mittlerer arterieller Druck

^a^Die jeweils erreichten Punkte der einzelnen Organsysteme werden addiert

Treffen bei der Sepsis trotz adäquater Volumensubstitution die beiden folgenden Bedingungen zu, spricht man von einem „septischen Schock“ [43]:

1. Vasopressoren sind erforderlich, um einen mittleren arteriellen Druck (MAP) von ≥ 65 mm Hg zu erreichen (beeinträchtigte Makrozirkulation);
2. Serumlaktat ≥ 2 mmol/l (≥ 18 mg/dl; beeinträchtigte Mikrozirkulation).

Da die Kalkulation des SOFA-Scores laborchemische Befunde voraussetzt, ist dieser Score insbesondere der innerklinischen intensivmedizinischen Umgebung vorbehalten und damit die definitive Diagnosestellung einer Sepsis erst in der Klinik möglich. Um Patienten mit einem hohen Risiko für das Vorliegen einer Sepsis schneller identifizieren zu können, wurde der „Quick-SOFA(qSOFA)“-Score eingeführt. Dieser eignet sich aufgrund seiner niedrigen Sensitivität allerdings weniger zum Sepsisscreening als vielmehr zur prädiktiven Risikostratifizierung kritisch erkrankter Patienten [1, 9, 30, 43, 46]. Dennoch sollte differenzialdiagnostisch an eine Sepsis gedacht werden, wenn bei einem Infektionsverdacht mindestens 2 der folgenden Kriterien erfüllt sind:

1. Atemfrequenz (AF) ≥ 22/min,
2. Vigilanzminderung,
3. Hypotension ≤ 100 mm Hg systolisch.

Neben dem qSOFA existiert eine Reihe weiterer Frühwarnscores (Tab. 3), die allesamt eine hohe Sensitivität zur Identifikation kritischer Patienten, jedoch nur eine geringe Spezifität für die Detektion einer Sepsis gemeinsam haben. Prinzipiell erheben diese Scoringsysteme in unterschiedlichen Kombinationen überwiegend Parameter, die zum minimalen Notfalldatensatz (MIND) der Deutschen Interdisziplinären Vereinigung für Intensiv- und Notfallmedizin (DIVI) zählen und regelhaft in der prähospitalen Notfallmedizin erfasst werden. Darüber hinaus erfassen manche Scores weitere Patienten‑, Anamnese- oder Therapiefaktoren oder aber bestimmte Laborparameter. Insbesondere wenn Blutbild oder klinische Chemie zur Errechnung eines Scores erforderlich sind, limitiert dies die Anwendungsmöglichkeit auf die Notaufnahme. Das Vorhandensein bzw. die Ausprägung der einzelnen Items ergibt innerhalb des jeweiligen Scoringsystems eine bestimmte Punktzahl, die ab einem definierten Wert einen potenziell kritischen Patienten identifiziert. Hierunter ist nach Ansicht der Autoren insbesondere der im Vereinigten Königreich etablierte „national early warning score (NEWS) 2“ hervorzuheben, der zur Detektion einer Sepsis im notfallmedizinischen Setting dem qSOFA-Score überlegen ist [26]. Der NEWS 2 impliziert ab einem kumulierten Absolutwert von ≥ 5 Punkten eine besondere Vigilanz und dessen Veränderung eignet sich darüber hinaus zur Verlaufsbeobachtung [34]. Häufig bilden die Scoringsysteme weitere Hinweise für das Vorliegen einer Infektion, wie z. B. Herzfrequenz, Hypo- oder Hyperthermie, ab.

**Table 3:** 

	SIRS	SOFA	qSOFA	[M/N] EWS	PRESEP	BAS	[M]RST	MEDS	CIS	PRESS	EWRS
Patientenfaktoren	**–**	**–**	**–**	**–**	**–**	**–**	**–**	**×**	**×**	**×**	**–**
Anamnesekriterien	**–**	**–**	**–**	**–**	**–**	**–**	**×**	**×**	**–**	**–**	**–**
Therapiefaktoren	**–**	**×**	**–**	**[×]**	**–**	**–**	**–**	**–**	**–**	**–**	**–**
*Vitalparameter*											
Atemfrequenz	**×**	**–**	**×**	**×**	**×**	**×**	**×**	**×**	**×**	**–**	**×**
Sauerstoffsättigung	**–**	**–**	**–**	**×**	**×**	**×**	**[×]**	**–**	**×**	**×**	**–**
Herzfrequenz	**×**	**–**	**–**	**×**	**×**	**–**	**×**	**×**	**×**	**–**	**×**
Blutdruck	**–**	**×**	**×**	**×**	**×**	**×**	**[×]**	**–**	**×**	**×**	**×**
Bewusstsein	**–**	**×**	**×**	**×**	**×**	**–**	**×**	**×**	**×**	**–**	**–**
Körpertemperatur	**×**	**–**	**–**	**×**	**×**	**–**	**×**	**×**	**–**	**×**	**×**
*Laborparameter*											
Blutzucker	**–**	**–**	**–**	**–**	**×**	**–**	**×**	**–**	**–**	**–**	**–**
Blutgasanalyse	**×**	**×**	**–**	**–**	**–**	**–**	**[×]**	**–**	**–**	**–**	**×**
Blutbild	**×**	**×**	**–**	**–**	**–**	**–**	**–**	**×**	**–**	**–**	**×**
Klinische Chemie	**–**	**×**	**–**	**–**	**–**	**–**	**–**	**–**	**–**	**–**	**–**

*SIRS* systemisches inflammatorisches Responsesyndrom [8, 22], *SOFA* „sequential organ failure assessment“ [48], *qSOFA* „quick SOFA“ [1, 9, 43, 46], [*M/N]EWS* „[modified/national] early warning score“ [34], *PRESEP* „prehospital early sepsis detection“ [5], *BAS* Blutdruck, Atmung, Sauerstoffsättigung 90 – 30 – 90 [5], [*M]RST* „[modified] Robson screening tool“ [5], *MEDS* „mortality in emergency department sepsis“ [42], *CIS* „cellular injury score“ [27], *PRESS* „prehospital recognition of severe sepsis“ [29], *EWRS* „early warning and response system“ [47]

^a^Eckige Klammern besagen, dass dieses Item nicht in alle der in der Spalte genannten Scores eingeht

Bei positivem Score oder wenn trotz negativem Scoring weiterhin eine Sepsis vermutet wird, soll nach Klinikaufnahme der SOFA-Score erhoben werden [9]. Die aktualisierten „surviving sepsis campaign“ (SSC)-Guidelines empfehlen nicht mehr die Nutzung des qSOFA, sondern Scoringsysteme wie MEWS bzw. NEWS (Tab. 3 [32]).

### Praxistipp.

Gerade auch prähospital können und sollten bei den verwendeten Scores (mit Ausnahme der Laborbefunde) alle Parameter erhoben und dokumentiert werden*.*

## Therapieprinzipien

Aufgrund des mitunter fulminanten Verlaufs und der vitalen Bedrohung einerseits und einer langen Dauer bis zum Vorliegen der mikrobiologischen Befunde andererseits müssen bei einer Sepsis diagnostische und therapeutische Schritte unverzüglich und parallel erfolgen. Diese folgen den Empfehlungen der S3-Leitlinie zur Sepsis [9], die sich wiederum an den SSC-Guidelines aus dem Jahr 2017 orientiert, welche kürzlich aktualisiert wurden [32] und und das Vorgehen in „Bündeln“ („bundles“) zusammenfassen.

### Merke.

In akut vital bedrohlichen Situationen hat sich das ursprünglich der Traumaversorgung entstammende „ABCDE-Schema“ bewährt.

**Figure 2:**
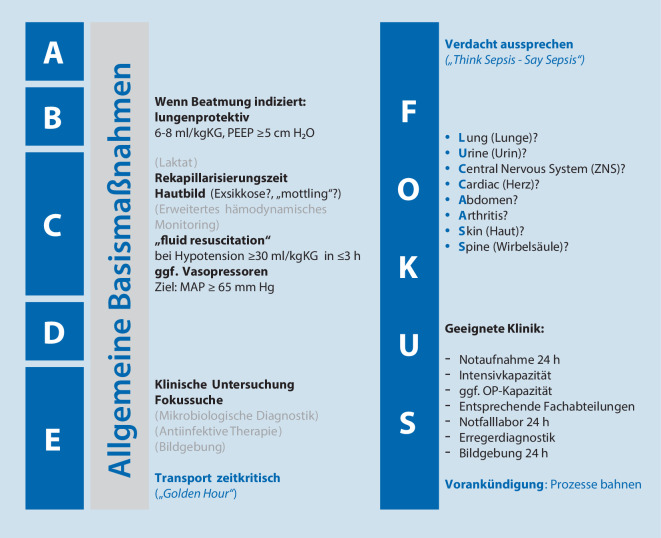

Da sich diese Therapieprinzipien in vielen kritischen Situationen der Notfallmedizin fest etabliert haben und jedem notfallmedizinisch Tätigen geläufig sein sollten, verweisen wir an dieser Stelle auf das Vorgehen und die Priorisierung gemäß *ABCDE-Schema*. Spezifische Punkte innerhalb dieses Schemas, die bei der Versorgung von Patienten mit (Verdacht auf) Sepsis über die allgemeinen Basismaßnahmen hinausgehen, werden in Abb. 2 hervorgehoben.

## Anamnese

Insbesondere bei erforderlicher Analgosedierung oder ggf. Narkoseeinleitung und Intubation gehören die Einsatzkräfte zu den letzten Personen, die den Patienten möglicherweise noch in einem ansprechbaren Zustand antreffen. Deshalb sollte, in zeitlich vertretbarem Umfang, die Anamnese anhand eines Schemas erhoben und dokumentiert werden.

Anamnestisch sollte unter anderem jegliche Form von Immunsuppression (als Folge von Vorerkrankungen, Alter sowie iatrogen oder hereditär) erfragt werden, stellt sie doch einen besonderen Risikofaktor für die Entwicklung einer Infektion und Sepsis dar.

## A und B: Atemwege und (Be‑)Atmung

Insbesondere bei pulmonalem Fokus kann eine Sauerstoffgabe, bei zunehmender respiratorischer Erschöpfung eine nichtinvasive Ventilation oder gar die endotracheale Intubation und kontrollierte Beatmung erforderlich werden. Das Vorgehen folgt der Handlungsempfehlung zur prähospitalen Notfallnarkose [6] und der S1-Leitlinie zum prähospitalen Atemwegsmanagement [45].

### Praxistipp.

Bei ausgeprägter Schocksymptomatik mit gestörter Mikrozirkulation misst die periphere Sauerstoffsättigung (S_p_O_2_) unter Umständen keine validen Werte.

Die Indikation für eine invasive Beatmung sollte kritisch geprüft werden, um die „on-scene time“ nicht zu verlängern und die klinische Behandlung der Infektion nicht zu verzögern. Andererseits darf dies jedoch nicht dazu führen, dass bei bestehender Indikation eine Intubation unterbleibt und ein Patient die Notaufnahme hypoxisch erreicht. Klare Indikationen zur prähospitalen Notfallnarkose und Atemwegssicherung, die auch für die Sepsis gelten, sind [6]:

- akute respiratorische Insuffizienz (Hypoxie oder Atemfrequenz < 6 oder > 29/min) bei Kontraindikationen zur bzw. Versagen der nichtinvasiven Ventilation;
- Bewusstlosigkeit oder neurologisches Defizit mit Aspirationsgefahr.

Der Vollständigkeit halber sei an dieser Stelle erwähnt, dass die Handlungsempfehlung zur prähospitalen Notfallnarkose zudem das schwere bzw. Polytrauma mit hämodynamischer Instabilität (Blutdruck < 90 mm Hg systolisch), Hypoxie (S_p_O_2_ < 90 % trotz O_2_-Gabe) oder Schädel-Hirn-Trauma (GCS < 9) als Intubationsindikation nennt [6].

Wird zur Sicherung der Atemwege und Beatmung eine Intubation und damit eine Narkoseeinleitung erforderlich, sind das Risiko einer Hypotension und Hypoxie sowie die prähospital erschwerten Umgebungsbedingungen zu bedenken. Die Atemwegssicherung muss zeitkritisch und zwingend erfolgreich durchgeführt werden [45].

### Praxistipp.

Propofol sollte als Induktionshypnotikum aufgrund seiner kreislaufdepressiven Wirkung bei hämodynamisch instabilen Patienten unbedingt vermieden werden. Auch Etomidat ist aufgrund der Suppression von Kortisol- und Aldosteronsynthese in diesen Fällen nicht geeignet. Die Autoren empfehlen die Narkoseinduktion, so sie denn erforderlich werden sollte, mit Esketamin, Midazolam, gegebenenfalls ergänzt um ein Opioid, sowie einem schnell anschlagenden Muskelrelaxans.

Die kontrollierte Beatmung folgt dem Konzept der „lungenprotektiven Beatmung“ mit Tidalvolumina von 6–8 ml/kgKG bei endinspiratorischem Atemwegsdruck von ≤ 30 cm H_2_O bzw. „driving pressure“ ≤ 15 cm H_2_O sowie einem PEEP von ≥ 5 cm H_2_O [9].

## C: Kreislauf

Die hämodynamische Stabilisierung nimmt bei Patienten mit (vermuteter) Sepsis bereits im prähospitalen Setting einen zentralen Stellenwert ein [9, 15].

### Praxistipp.

Solange keine invasive arterielle Blutdruckmessung erfolgen kann, ist unbedingt auf engmaschige (Messintervall < 5 min) Kontrollen von Blutdruck und Rekapillarisierungszeit zu achten, um zuverlässig einen MAP von ≥ 65 mm Hg bzw. systolischen Blutdruck ≥ 100 mm Hg aufrechterhalten zu können. Hierzu sind in der Regel mehrere intravenöse (i.v.-)Zugänge erforderlich. Ist dies durch Schocksymptomatik, Gefäßsituation oder Ödembildung absehbar nicht realisierbar, kann auch ein intraossärer (i.o.-)Zugang geschaffen werden. Hierbei ist allerdings zu beachten, dass der Fluss über einen i.o.-Zugang erfahrungsgemäß meist nicht für eine Volumentherapie im Sinne der im Folgenden vorgestellten „fluid resuscitation“ ausreicht und damit die „C-Problematik“ allein nicht löst. In der Klinik wird in der Regel ein zentralvenöser Zugangsweg mit großen Lumina etabliert. Auf die Sinnhaftigkeit einer vorübergehenden Applikation von Vasopressoren über periphere i.v.-Zugänge wird in den neuen SSC-Guidelines explizit hingewiesen, um den Beginn der vasoaktiven Therapie (und die dadurch erhoffte Kreislaufstabilisierung) nicht bis zur Anlage eines zentralen Venenkatheters zu verzögern [15].

Neben Zeichen einer Exsikkose interessiert beim Hautbefund das Vorliegen einer Marmorierung („mottling“) an Knie- und Ellenbogengelenk, auf die bereits prähospital geachtet werden sollte. Die Marmorierung ist Abbild der Mikrozirkulationsstörung, die Ausprägungsschwere stellt einen negativen Prognoseprädiktor dar [2].

### Praxistipp.

Mit der zunehmenden Verbreitung portabler Ultraschallgeräte im Notarztdienst kann der kardiozirkulatorische Status auch sonographisch („point of care ultrasound“, POCUS) schnell und zuverlässig abgeschätzt und im Verlauf kontrolliert werden.

### Laktatmessung

Die Messung des Laktats im Serum dient als Surrogatparameter der Mikrozirkulation sowie als Ausgangspunkt zur weiteren Verlaufsbeobachtung und Steuerung der hämodynamischen Stabilisierung („resuscitation“) [15]. Die Aufnahme des Laktatwerts in die Definition des septischen Schocks wird häufig als Kritikpunkt angeführt, da die Messung dieses Laborparameters nicht immer und überall verfügbar ist [25]. Die „Point-of-Care“-Messung des Laktats kann mit einfachen Geräten und Teststreifen erfolgen und entspricht im Vorgehen der bereits etablierten Blutzuckermessung. Dies ist auch im Rettungsdienst prinzipiell möglich und aus Sicht der Autoren sinnvoll. Mit der Verfügbarkeit portabler Blutgasanalysegeräte können bereits am Einsatzort eine vollständige Blutgasanalyse und über Laktat und Blutzucker hinaus auch Sauerstoff- bzw. Kohlendioxidpartialdrücke sowie der Hämoglobingehalt gemessen und hierdurch differenzialdiagnostisch und therapeutisch bedeutsame Hinweise bei kritischen Patienten geliefert werden.

Die ANDROMEDA-SHOCK-Studie konnte zeigen, dass die Steuerung der Hämodynamik anhand der Rekapillarisierungszeit („capillary refill time“) verglichen mit der Steuerung nach den Laborwerten des Serumlaktats hinsichtlich der Überlebensraten zumindest gleichwertig und bezogen auf die Häufigkeit von Organversagen sogar überlegen ist. Demzufolge könnte im prähospitalen Setting die Volumentherapie anhand des einfach zu erhebenden Markers „Rekapillarisierungszeit“ evaluiert werden, wobei das Ziel das Erreichen einer Rekapillarisierungszeit von < 3 s darstellt. Bei der Interpretation dieser Studienergebnisse ist allerdings zu berücksichtigen, dass diese für die Detektion eines Überlebensvorteils von absolut 15 % bei diesem komplexen Krankheitsbild „underpowered“ erscheint [17]. Konsequenterweise empfehlen die aktualisierten SSC-Guidelines explizit die (ergänzende) Nutzung der Rekapillarisierungszeit zur Steuerung der Volumentherapie [32].

#### Merke.

Prähospital kann anstelle des Laktats auch die Rekapillarisierungszeit zur Beurteilung der Mikrozirkulation herangezogen werden.

### „Fluid resuscitation“

Zur Stabilisierung der Hämodynamik wird bei hypotensiven Kreislaufverhältnissen ein Bolus von 30 ml/kgKG balancierter Vollelektrolytlösung innerhalb der ersten 3 h appliziert [15, 37].

#### Praxistipp.

Die „fluid resuscitation“ kann bereits prähospital begonnen werden, wenn eine Sepsis die naheliegendste Diagnose ist und kein Anhalt für eine Volumenüberladung besteht.

Die reine „physiologische“ Kochsalzlösung (NaCl 0,9 %) ist obsolet und unter den kolloidalen Infusionslösungen ist Hydroxyethylstärke kontraindiziert. Andere osmolar wirksame Infusionslösungen (Gelatine, Albumin) sind allenfalls in einer späteren Phase sinnvoll, falls sich der Patient mit balancierten kristalloiden Infusionslösungen und Katecholaminen hämodynamisch nicht stabilisieren lässt. Weitere Flüssigkeitsgaben können in Erwägung gezogen werden, wenn weiterhin Hinweise für eine Hypoperfusion vorliegen. Im prähospitalen Setting können hierfür unter anderem Herzfrequenz, Blutdruck, Rekapillarisierungszeit, Sauerstoffsättigung und Atemfrequenz herangezogen werden; ein darüber hinausgehendes erweitertes hämodynamisches Monitoring ist prähospital nicht regelhaft verfügbar.

Bei manifestem Schock ist in der Regel neben der Flüssigkeitstherapie auch der frühzeitige Einsatz von Vasopressoren erforderlich, um einen MAP von 65 mm Hg schnellstmöglich zu erreichen und damit die Perfusion aufrechtzuerhalten.

#### Praxistipp.

Das Katecholamin der Wahl ist in diesem Fall Noradrenalin, beginnend mit Boli à 10 µg; im Verlauf scheint auch prähospital die Etablierung einer Spritzenpumpe sinnvoll.

Sollte hierdurch keine ausreichende hämodynamische Stabilität erreicht werden, können zusätzlich Adrenalin oder das prähospital meist nicht verfügbare Vasopressin sowie in einem weiteren Eskalationsschritt Dobutamin eingesetzt werden. Bei letzterem ist eine periphere Vasodilatation zu bedenken, die ausgeprägte Erhöhung des Herzzeitvolumens bei septischer Kardiomyopathie ist hauptsächlich auf eine Zunahme der Herzfrequenz zurückzuführen [9, 15, 37].

#### Merke.

Zur Kreislaufstabilisierung werden balancierte kristalloide Infusionslösungen verwendet und zur Aufrechterhaltung der Perfusion ggf. frühzeitig um Katecholamine (Noradrenalin) ergänzt.

## D: neurologischer Status

Der neurologische Status einschließlich Blutglukose ist prähospital auch bei der Sepsis unbedingt zu erheben und sorgfältig zu dokumentieren, sodass Veränderungen im Verlauf bemerkt werden können. Parameter wie GCS und Blutglukosekonzentration gehen zudem in unterschiedliche diagnostische Scores ein (Tab. 2 und 3).

## E: Exploration und Umgebungsbedingungen

Die während der prähospitalen Versorgungsphase erhobenen Untersuchungsbefunde einschließlich Körpertemperatur und Hautbefund (z. B. Exsikkose, „mottling“ – s. oben) können ebenso wie die beobachteten Umgebungsbedingungen wertvolle Hinweise für das weitere Vorgehen liefern.

### Mikrobiologische Diagnostik

Zur mikrobiologischen Diagnostik gehört die Abnahme von Blutkulturen und je nach vermutetem Fokus ggf. die erweiterte Erregerdiagnostik, beispielsweise im Urin, respiratorischen Sekret, Liquor oder in Abstrichen. Dies soll grundsätzlich vor Beginn einer antimikrobiellen Therapie erfolgen, diese jedoch auch nicht verzögern [9, 15]. Die Gewinnung mikrobiologischer Proben ist im Rettungsdienst prinzipiell möglich [49], setzt jedoch streng sterile Bedingungen voraus. Als weiteres Problem werden beispielsweise Blutkulturen nur in 2,6 % der Rettungsdienstbereiche überhaupt vorgehalten [11] und die prähospital genutzten Produkte könnten möglicherweise nicht mit denen der Klinik übereinstimmen und damit das weitere prozedurale Verfahren beeinflussen. Dies schränkt die Fokussuche (Tab. 1) häufig auf eine sorgfältige Anamnese und körperliche Untersuchung ein und damit auch die Wahrscheinlichkeit, eine Sepsis an der Einsatzstelle als solche zu erkennen. Die Limitationen im prähospitalen Bereich erklären jedoch nicht, weshalb bei jedem 5. Patienten mit Sepsis in Deutschland überhaupt keine Blutkultur abgenommen wird. Nur etwa ein Drittel aller bei Sepsis entnommenen Blutkulturen liefert tatsächlich auch einen mikrobiologischen Befund (Tab. 4, [39]).

**Table 4:** 

Erreger	Häufigkeit^a^ in %
Gramnegative Bakterien – *Escherichia coli* – Bacteroides species – *Pseudomonas aeruginosa* – *Klebsiella pneumoniae* – Enterobacter species	33–73
Grampositive Bakterien – Enterococcus species – Koagulasenegative Staphylokokken – *Staphylococcus aureus* – *Streptococcus viridans* – *Clostridium difficile*	23–56
Pilze, Hefen	4–26
– Candida species	
– *Aspergillus fumigatus*	
Anaerobier	3–4
– *Clostridium difficile*	
– *Bacteroides fragilis*	
Viren	2–3
– Cytomegalovirus	
– Influenza	
– Herpes-simplex-Virus	
– Adenovirus	
– Respiratorisches Synzytial-Virus	
– Coronavirus	
Legionellen	< 1
– *Legionella pneumophila*	
Mykobakterien	< 1
– *Mycobacterium tuberculosis*	

^a^Die Häufigkeitsangaben nach [13, 14, 20, 39] beziehen sich auf Intensivstationen in Deutschland. Da nicht jeder Patient mit Sepsis aus der Notaufnahme auf eine Intensivstation verlegt wird, lassen sich die Häufigkeiten nicht uneingeschränkt auf das prähospitale Setting übertragen

Außerdem ist zu berücksichtigen, dass nicht nur Bakterien, sondern z. B. auch Pilze und Viren (als aktuelles Beispiel das Coronavirus SARS-CoV-2) eine Sepsis verursachen können. Je nach vermutetem Erreger ist ggf. eine Spezialdiagnostik erforderlich.

### Antiinfektive Therapie

Die Auswahl der kalkulierten antiinfektiven Therapie bei unklarem Fokus richtet sich nach klinischer Symptomatik, antimikrobieller Vorbehandlung sowie Resistenzlage und soll so schnell wie möglich erfolgen [7, 33]. Jede Stunde, die der Beginn der Antibiose verzögert wird, erhöht die Mortalität der Sepsis [21, 40]. Ob der Beginn einer antiinfektiven Therapie bereits am Einsatzort im Hinblick auf das Behandlungsergebnis der Patienten sinnvoll ist, wird kontrovers diskutiert. So konnten einige Pilot- bzw. Machbarkeitsstudien zeigen, dass die Abnahme von Blutkulturen und die Antibiotikagabe sowohl in auf „paramedics“ basierten [49] als auch in notarztbasierten Systemen praktikabel sind und einen Zeitvorteil bieten können [12, 38]. So werden in 26 % der deutschen Rettungsdienstbereiche Antibiotika vorgehalten; überwiegend Cephalosporine und zu einem deutlich geringeren Anteil Penicilline oder sonstige Wirkstoffgruppen [11].

Ob der prähospitale Beginn einer antiinfektiven Therapie auch mit höheren Überlebensraten einhergeht, lässt sich derzeit noch nicht abschließend beantworten. Die „PHANTASi-Studie“ war die erste multizentrische randomisierte kontrollierte Studie, die den Nutzen einer bereits durch Rettungsfachpersonal begonnenen Antibiose bei 2698 Patienten mit vermuteter Sepsis in den Niederlanden untersuchte. Die prähospitale Applikation eines Cephalosporins der Gruppe 3 ging zwar mit einem Zeitvorteil von 26 min vor Eintreffen in der Notaufnahme (zuzüglich im Median 70 min bis zum Beginn der Antibiotikagabe), nicht aber mit einer geringeren Sterblichkeit einher. Diese betrug sowohl in der Interventions- als auch in der Kontrollgruppe 8 % nach 28 Tagen, was für die Sepsis eine extrem niedrige Mortalität darstellt und dadurch möglicherweise die Effektivität einer prähospitalen Antibiotikagabe verschleiern könnte [3]. Überlegungen, inwiefern dieses Ergebnis auch mit der Auswahl des Antibiotikums zusammenhängt und ob andere Wirkstoffgruppen (z. B. Acylaminopenicilline mit β‑Laktamase-Inhibitor oder Carbapenem) möglicherweise zu anderen Ergebnissen geführt hätten, bleiben rein spekulativ. Bei prähospitaler Gabe eines Antibiotikums kann die mikrobiologische Diagnostik am Einsatzort, wie im vorigen Abschnitt ausgeführt, mit Limitationen verbunden oder erst in der Klinik vollständig möglich sein. Eine Abnahme von Blutkulturen nach Beginn der Antibiose reduziert jedoch die ohnehin geringe Positivitätsrate nochmals massiv auf rund 27 % [36].

Auch in der aktuellen Version empfehlen die SSC-Guidelines den sofortigen Beginn der Antibiose, idealerweise innerhalb von einer Stunde nach Erkennen des Krankheitsbildes. Die Evidenz für diese Empfehlung ist jedoch niedrig (Septischer Schock) bzw. sehr niedrig (Sepsis ohne Schock) und die Daten stammen überwiegend aus amerikanischen Notaufnahmen, so dass diese nicht uneingeschränkt auf den prähospitalen Bereich übertragbar sind [15].

Eine Stellungnahme der European Society of Emergency Medicine (EuSEM) kritisiert die Fixierung auf ein starres „1 h bundle“: So sei zwar die frühe Antibiotikagabe eine der wenigen Maßnahmen innerhalb der Sepsisbündel, die mit der Mortalität korrelieren, doch solle gleichzeitig eine unnötige Antibiose (und Volumentherapie) vermieden werden. Ohne den Faktor Zeit in Abrede zu stellen, empfiehlt die EuSEM, mit der Antibiose erst dann zu beginnen, wenn die Diagnose „Sepsis“ die wahrscheinlichste ist, und das Ziel von einer Stunde ab Triage für sorgfältig selektierte Patienten anzustreben [17]. In einer aktuellen Empfehlung spricht sich auch die Infectious Diseases Society of America (IDSA) dementsprechend dafür aus, lediglich beim septischen Schock, nicht aber bei der Sepsis bereits innerhalb des „1 h bundle“ mit der antiinfektiven Therapie zu beginnen [32]. Insgesamt bleibt festzuhalten, dass die Evidenzlage für den Nutzen einer prähospitalen Antibiotikagabe aufgrund der Limitationen an der Einsatzstelle einerseits und nicht ausreichend aussagekräftiger Studien andererseits für eine abschließende Bewertung derzeit noch unzureichend ist. Weitere Studien werden die Frage beantworten müssen, ob der prähospitale Beginn einer antiinfektiven Therapie auch mit einem verbesserten Behandlungsergebnis einhergeht.

In Anlehnung an die „door-to-needle time“ beim ischämischen Schlaganfall bzw. „door-to-balloon time“ beim okklusiven Myokardinfarkt sollte bei der Sepsis durch die Schaffung entsprechender prä- und intrahospitaler Strukturen (beispielsweise ein „Nichttraumaschockraum“ mit angemessenen personellen Ressourcen, konkreten und praktikablen Standardarbeitsanweisungen [„standard operating procedure“, SOP] sowie regelmäßigem theoretischem und praktischem Training) nach präziser Voranmeldung eine möglichst kurze „door-to-antibiotic time“ erreicht werden [15, 28].

#### Merke.

Ein prähospitaler Beginn der antiinfektiven Therapie wird aufgrund der gegenwärtig limitierten Evidenzlage kontrovers diskutiert.

## Schnittstelle Notaufnahme

Eine effiziente Kommunikation, insbesondere an kritischen Schnittstellen wie der Notaufnahme, mit einer sorgfältigen und vollständigen Übergabe ist entscheidend, um die weiteren Behandlungspfade zu triggern. Hierdurch wird die adäquate Weiterversorgung mit gezielter Therapie und schnellstmöglicher Fokussanierung ermöglicht [15].

Um die Sepsis als solche erkennen zu können, müssen die für die Diagnosekriterien relevanten Befunde auch erhoben werden. Bedauerlicherweise werden diese wichtigen Parameter, die direkte therapeutische Konsequenzen haben und das weitere innerklinische Management beeinflussen können, zu einem erschreckend hohen Anteil nicht erhoben [10].

### Dokumentation

Das weiterversorgende Team der Notaufnahme bzw. Intensivstation steht im Verlauf häufig vor der Herausforderung, Patienten zu versorgen, über die nur wenig bekannt ist. Insbesondere wenn eine Analgosedierung oder ggf. Narkose und Beatmung erforderlich geworden sind, ist die Beschaffung von Informationen über den Patienten häufig mit einem hohen Aufwand verbunden. Deshalb kommt einer präzisen, sorgfältigen und umfassenden Dokumentation durch das prähospital versorgende Team ein besonderer Stellenwert zu.

#### Praxistipp.

Für die weiterbehandelnden Kollegen können neben der strukturierten Anamnese auch der Allgemeinzustand des Patienten vor dem Notfallereignis, die Kontaktdaten eines Angehörigen und ggf. eine vorliegende Vorsorgevollmacht und/oder Patientenverfügung hilfreich sein.

## Besonderheiten der intrahospitalen Notfallversorgung

Unmittelbar nach Klinikaufnahme erfolgt die Komplettierung der klinischen und mikrobiologischen Diagnostik. Je nach Fokus und erwartetem Erregerspektrum wird spätestens jetzt die kalkulierte antiinfektive Therapie begonnen [9].

### Fokussanierung

Das klinische „1 h bundle“ schließt mit der Evaluation einer möglichen Fokussanierung, die höchste Priorität genießt und sobald als logistisch und medizinisch möglich erfolgen soll [9, 15]. Anhand der Arbeitshypothese und des antizipierten Fokus sollte eine adäquate Zielklinik mit Bedacht gewählt und vorab informiert werden, um bereits die weiteren Behandlungspfade zu triggern und die Übernahme in einer geeigneten Umgebung (z. B. Aufnahmearbeitsplatz der Intensivstation oder Schockraum) vorbereiten zu können. Die Übergabe durch Notarzt bzw. Rettungsdienst an die weiterbehandelnde Klinik stellt eine kritische Schnittstelle dar, die jedoch für eine unmittelbare, strukturierte und gezielte Weiterversorgung der Patienten essenziell ist.

#### Merke.

Die Fokussanierung genießt höchste Priorität, sodass ein zeitkritischer Transport in eine geeignete Klinik erfolgen soll. Durch eine rechtzeitige Vorabinformation können die weiteren Behandlungspfade getriggert werden.

## Strukturelle Vorkehrungen

### Prävention

Die Einhaltung der Hygienestandards und multimodalen Strategien zur Infektionsprävention, darunter insbesondere die Compliance zur Händehygiene, spielen bei der Prävention der Sepsis im Rettungsdienst eine zentrale Rolle. Durch Impfungen entsprechend der Empfehlungen der Ständigen Impfkommission am Robert Koch-Institut kann die Ausbreitung von Infektionserkrankungen eingedämmt werden und es liegt an den Beschäftigten im Gesundheitssystem, zum Wohl der Patienten und auch zum Eigenschutz hier mit gutem Beispiel voranzugehen [44].

## Organisation und Ausbildung

Alle Einsatzkräfte sollten in Bezug auf Infektionsprävention und Früherkennung der Sepsis, wie dies bei anderen Entitäten wie der Reanimation oder dem Polytrauma bereits fest etabliert ist, regelmäßig theoretisch und praktisch fortgebildet werden [9, 15]. Das konkrete Vorgehen sollte strukturierten Algorithmen im Sinne von SOP folgen, die auf die prä- und intrahospitalen Ressourcen vor Ort abgestimmt sind. Obwohl hierfür allgemeine Handlungsanweisungen der Berufsverbände bzw. Behörden vorliegen [4, 31], nutzen nur 10 % der Rettungsdienstbereiche Algorithmen für die Therapie der Sepsis [11]. Im Rahmen des Qualitätsmanagements sollte die Versorgung von Patienten mit vermuteter oder manifester Sepsis als Tracer-Diagnose regelmäßig hinsichtlich der Versorgungsqualität, -zeiten, Voranmeldung und Übergabe an Schnittstellen ausgewertet und gegebenenfalls optimiert werden.

## Fazit für die Praxis

- Die Sepsis ist ein häufiges, vital bedrohliches und oft fulminant verlaufendes Krankheitsbild.
- Jeder Verdacht soll ausgesprochen und differenzialdiagnostisch berücksichtigt werden.
- Die Therapie ist zeitkritisch und folgt dem Prinzip der „golden hour“.
- Prähospitale Schwerpunkte:
  - Sorgfältige Anamnese und Untersuchung.
  - Stabilisierung der Vitalparameter nach „ABCDE-Schema“.
  - Falls indiziert Notfallnarkose, Atemwegssicherung und lungenprotektive Beatmung.
  - Volumentherapie mit balancierter Vollelektrolytlösung (30 ml/kgKG in 3 h) nach Blutdruck (MAP ≥ 65 mm Hg bzw. systolisch ≥ 100 mm Hg; primär) und Rekapillarisierungszeit (< 3 s) bzw. Laktat (< 2 mmol/l; sekundär) gesteuert.
  - Häufig sind zur hämodynamischen Stabilisierung zusätzlich Katecholamine erforderlich.
  - Die Auswahl einer geeigneten Klinik und Voranmeldung soll weitere Behandlungspfade triggern und eine gezielte Therapie sowie schnellstmögliche Fokussanierung ermöglichen.
- Die Gewinnung mikrobiologischer Proben und Einleitung der antiinfektiven Therapie sind prähospital prinzipiell machbar, wenngleich diese nicht flächendeckend vorgehalten werden und die Evidenzlage limitiert ist.
- Wie bei allen häufigen und bedrohlichen Notfällen ist regelmäßiges Training wichtig.

## Abkürzungen

AF: Atemfrequenz
AZ: Allgemeinzustand
BAS: Blutdruck, Atmung, Sauerstoffsättigung (90 – 30 – 90)
CIS: „Cellular injury score“
EuSEM: European Society of Emergency Medicine
EWRS: „Early warning and response system“
EWS: „Early warning score“
GCS: Glasgow Coma Scale
i.v.: Intravenös
i.o.: Intraossär
IDSA: Infectious Diseases Society of America
k. A.: Keine Angabe
kgKG: Kilogramm Körpergewicht
MAP: Mittlerer arterieller Druck
MEDS: „Mortality in emergency department sepsis“
MEWS: „Modified early warning score“
MRST: „Modified Robson screening tool“
NEWS: „National early warning score“
PEEP: „Positive end-exspiratory pressure“
PRESEP: „Prehospital early sepsis detection“
PRESS: „Prehospital recognition of severe sepsis“
qSOFA: „Quick sequential organ failure assessment“
RST: „Robson screening tool“
SARS-CoV‑2: „Severe acute respiratory syndrome coronavirus 2“
SIRS: Systemisches inflammatorisches Responsesyndrom
SOFA: „Sequential organ failure assessment“
SOP: „Standard operating procedure“
S_p_O_2_: Periphere Sauerstoffsättigung
SSC: „Surviving sepsis campaign“
ZNS: Zentrales Nervensystem
